# Species Composition of a Small Mammal Community and Prevalence of *Echinococcus* spp. in the Alpine Pastoral Area of the Eastern Tibetan Plateau

**DOI:** 10.3390/pathogens13070558

**Published:** 2024-07-02

**Authors:** Jia-Xin Zheng, Xiao-Hui Sun, Xu Wei, Gang Wang, Chang-Qing Yuan, Xiao-Dong Weng, Qing-Qiu Zuo, Jia-Yu Liu, Zhi-Qiang Mu, Tian-Ci Mao, You-Zhong Ding, Xiao-Ming Wang, Xu Wang, Zheng-Huan Wang

**Affiliations:** 1 School of Life Sciences, East China Normal University, Shanghai 200241, China; 2 Institute of Biological Resources, Jiangxi Academy of Sciences, Nanchang 330096, China; 3 National Key Laboratory of Intelligent Tracking and Forecasting for Infectious Diseases, National Institute of Parasitic Diseases, Chinese Center for Disease Control and Prevention (Chinese Center for Tropical Diseases Research), Shanghai 200025, China; 4 Key Laboratory of Parasite and Vector Biology, National Health Commission of the People’s Republic of China, Shanghai 200025, China; 5 World Health Organization Collaborating Center for Tropical Diseases, Shanghai 200025, China

**Keywords:** small mammals, *Echinococcus multilocularis*, *E. shiquicus*, prevalence, species composition

## Abstract

We aimed to investigate the species composition of a small mammal community and the prevalence of *Echinococcus* spp. in a typical endemic area of the Tibetan Plateau. One pika and five rodent species were identified based on the morphological characteristics of 1278 small mammal specimens collected during 2014–2019. Detection of *Echinococcus* DNA in tissue samples from small mammal specimens revealed that *Ochotona curzoniae* (pika, total prevalence: 6.02%, 26/432), *Neodon fuscus* (5.91%, 38/643), *N. leucurus* (2.50%, 3/120), and *Alexandromys limnophilus* (21.74%, 10/46) were infected by both *E. multilocularis* and *E. shiquicus*; *Cricetulus longicaudatus* (16.67%, 1/6) was infected by *E. shiquicus*; and no infection was detected in *N. irene* (0/15). *Neodon fuscus* and *O. curzoniae* were the two most abundant small mammal species. There was no significant difference in the prevalence of pika and the overall rodent species assemblage (6.26%, 53/846); however, the larger rodent populations suggested that more attention should be paid to their role in the transmission of echinococcosis in the wildlife reservoir, which has long been underestimated. Moreover, although DNA barcoding provides a more efficient method than traditional morphological methods for identifying large numbers of small mammal samples, commonly used barcodes failed to distinguish the three *Neodon* species in this study. The close genetic relationships between these species suggest the need to develop more powerful molecular taxonomic tools.

## 1. Introduction

Small mammals, mainly lagomorphs and rodents, comprise the most abundant group of mammals, with the largest number of individuals and the widest distribution [1]. They contribute to the energy flow and material cycle in ecosystems and the dispersal of plant seeds, as well as enhancing biodiversity [2]. Despite maintaining ecosystem stability and biodiversity [3], however, they are blamed for their impacts on natural ecosystems and human society; for instance, small mammals were blamed for the degradation of the grassland ecosystem in western China [4,5]. Small mammals are also hosts of many zoonotic diseases, which can be transmitted to humans directly or indirectly through various methods, including predation and excretion [6]. Small mammals have been widely reported to be susceptible to leptospirosis [7], coronaviruses [8], hemorrhagic fever [9], echinococcosis [10], plague, and other diseases [11]. Understanding the population ecology of small mammals thus provides fundamental information relevant to the conservation biology and epidemiology of zoonotic diseases.

The alpine meadow pastoral area of the eastern part of the Tibetan Plateau in China has been identified as a hot spot for echinococcosis, with the highest prevalence worldwide [12]. Echinococcosis comprises a group of zoonotic diseases caused by the larvae of *Echinococcus* species, which are transmitted between canids (as definitive host species) and herbivores (as intermediate host species). Three *Echinococcus* species have been identified in this area: *Echinococcus granulosus s.l.* is transmitted between large herbivores and canids, while *E. multilocularis* and *E. shiquicus* are transmitted between small mammals and canids. Among these three species, *E. granulosus* and *E. multilocularis* larvae can infect humans and cause echinococcosis. Hydatic echinococcosis caused by *E. multilocularis* is the most severe type of echinococcosis, with a fatality rate without medical intervention > 90% [13]. Because of its significant risks to human livelihood in the vast pastoral areas in western China, echinococcosis has been listed as a major infectious disease eligible for free treatment since 2007 [14]. Small mammals are widely distributed in western China and serve as intermediate hosts of *E. multilocularis* and *E. shiquicus*; however, the taxonomy of small mammal communities in west China, including the Tibetan Plateau, remains largely unknown [15]. Even among local small mammal communities with known species composition, information on the prevalence of *Echinococcus* species and their dynamics in these putative host species is still limited [10]. Moreover, population trends of host species in small mammal communities are variable, and even within species the dynamics of local populations can differ remarkably because of specific differences in population and environmental characteristics [16]. Understanding the species composition and population dynamics of these small mammals is thus fundamental for monitoring and controlling echinococcosis in endemic areas [17,18].

The distribution and transmission of the above-mentioned three *Echinococcus* species in the chosen study area in Shiqu County, a typical endemic area located in the eastern part of the Tibetan Plateau, have been confirmed [19,20]. Notably, however, although small mammal species are abundant, information on their ecology and biology is still limited [13,15,21]. Recent taxonomic studies, including morphological and phylogenetic methods, have been conducted on small mammals in western China, including the Tibetan Plateau and contiguous areas [22,23,24,25,26], and the taxonomic and phylogenetic statuses of key species in this vast area have been re-evaluated, e.g., [22,27,28,29,30]. The taxonomic information derived from these studies has improved our understanding of the species composition and population dynamics in Shiqu County. This study therefore aimed to investigate the species composition of the small mammal community in Shiqu County and evaluate the prevalence of *Echinococcus* spp. and the population dynamics of its intermediate host species. We also considered the efficiencies of different taxonomic methods for host species identification during large-scale screening for epidemiological purposes. Ultimately, the results will help to provide basic information and identify suitable methodologies for the surveillance, prevention, and control of echinococcosis from the perspective of the population ecology of small mammals in endemic areas.

## 2. Materials and Methods

### 2.1. Study Area and Specimen Collection

The study was carried out in Yongbo Gou (33°11′ N, 97°39′ E), Shiqu County, Ganzi Tibetan Autonomous Prefecture, Sichuan Province, which is a typical pastoral area located in the eastern Tibetan Plateau, with an average elevation > 4200 m. The primary vegetation type is alpine meadow, the dominant herbaceous species being *Kobrecia parvula* and *K. setchwanensis* and the dominant shrub species being mainly *Salix takasagoalpina* and *Potentilla fruticosa*. During the warm season (June to September), July and August are the best months to capture small mammals, with lush vegetation and the highest daily temperatures [31].

Small mammals were collected from July to August in 2014, 2015, 2016, and 2019, during the annual wildlife plague (*Yersinia pestis*) surveillance organized by the Shiqu County Center for Disease Control (Shiqu CDC). Quadrats measuring 50 m × 50 m were set randomly and 400 back-broken traps were placed at active den entrances in each quadrat before 10:00. Trapped small mammals were collected at 17:00 on the same day and 9:00 the following day, respectively. The specimens were processed as described by Wang et al. [10]. Briefly, each individual was sexed and measured, and the head was stored in a 50 mL capped tube with 95% ethanol for further species identification. The body was then dissected to check for *Echinococcus* infection. If lesions were detected, a small portion was removed, and a piece of liver tissue was removed from individuals without obvious lesions. All lesion and liver samples were stored separately in 2 mL storage tubes with 95% ethanol at −20 °C.

### 2.2. Morphological Species Identification

The small mammal community in Shiqu County is mainly composed of *Ochotona curzoniae* (plateau pika) and several rodent species belonging to the family Circetidae [10]. Although the morphological criteria for *O. curzoniae* are clear [32], the efficient and reliable identification of the closely related rodent species in large numbers of specimens remains a challenge for epidemiological studies. The identification of small mammal species was based on quantitative morphological measurements and qualitative characteristics, including pelage color patterns and craniodental characteristics, especially of the molars [33,34,35]. The collected specimens were also compared with specimens in the museum of the Northwest Institute of Plateau Biology, Chinese Academy of Sciences, for additional confirmation. 

We evaluated the morphological similarities among detected rodent species using a quantitative morphology algorithm, including data for 8 external and 12 craniodental measurements using principal component analysis (PCA), with SPSS v22.0 [36]. The eight external measurements were bodyweight, ear length, head–body length, shoulder height, tail length, forefoot length, hindleg length, and hindfoot length. To collect craniodental data, the preserved head of each rodent individual was cleaned by mealworms (*Tenebrio molitor*) [37] and 12 craniodental characteristics were then measured, including greatest skull length, zygomatic breadth, skull height, rostrum breadth, orbital length, length of maxillary toothrow, length of mandibular toothrow, breadth of first maxillary molars, external alveolar breadth, length of incisive foramen, breadth of incisive foramen, and breadth of braincase [38,39]. With PCA, the varimax rotation algorithm was used during the matrix calculation, and principal components with eigenvalues ≥ 1 were selected for final two-dimensional spatial distribution presentations. 

### 2.3. Molecular Species Identification

Most of the rodent specimens collected in Shiqu County were vole species [10]. Liu et al. reported that vole species of the Arvicolini tribe from the Tibetan area are usually closely related taxonomically [22]. DNA barcoding is a powerful and efficient modern tool for species identification. To further test the accuracy and efficiency of this molecular taxonomic technique, we developed a barcoding species identification protocol using rodent specimens collected in 2014 and 2016 and compared the taxonomic results from barcoding with those from traditional morphological identification.

Total DNA was extracted from muscle tissue samples from preserved heads of trapped rodent individuals using a TIANamp Genomic DNA kit (Tiangen, Beijing, China) according to the manufacturer’s protocol. The mitochondrial cytochrome b (*Cytb*) and cytochrome oxidase I (*COI*) genes, the nuclear β-fibrinogen (*FGB*) gene, and the growth hormone receptor (*GHR*) gene were selected as multiple DNA barcodes for polymerase chain reaction (PCR) amplification for species identification for each rodent sample. The primer information is provided in Table S1. PCR amplification was performed in a 25 μL reaction with 1 μL of each primer (10 μmol/L; Sangon Biotech, Shanghai, China), 1 μL DNA, 0.5 μL 2% bovine serum albumin (Takara, Beijing, China), 12.5 μL Premix Taq (Takara), and 9 μL RNase-free water (Takara). The PCR conditions for each of the four genes are listed in Table S2. All amplified PCR products were visualized by 1% agarose gel electrophoresis and sent to Shanghai Sangon Biotech for sequencing.

The PCR-amplified sequences and GenBank sequences for relevant rodent species (accession numbers shown in Table S3) were aligned in MEGA v7 [40]. Nucleotide and haplotype diversities were analyzed using DNASP v5 [41] after confirming the unsaturation of base substitution in DAMBE v5 [42]. The optimal nucleotide substitution model was selected according to the Akaike Information Criterion calculated by jModelTest v2.1.7 [43]. Maximum likelihood (ML) trees were constructed using MEGA v7 with 1000 bootstrap replications. Bayesian trees were constructed using MrBayes v3.2.7 [44] by setting the Markov chain Monte Carlo posterior probability estimation for 2,000,000 generations with 1000-generation sampling intervals, and the first 25% of aging samples were discarded when summing up each tree. If the average standard deviation of the split frequency after the 2,000,000-generation calculation was still >0.01, an extra 100,000-generation calculation was added until the value was <0.01. The final phylogenetic trees were edited and presented using FigTree v1.4.3 [45].

We further tested the genetic divergence of rodent species in Shiqu County by analyzing the population genetic structure of identified rodent species based on sequential data for the four gene loci from our specimens. Inter- and intraspecific genetic distances were calculated using the Kimura 2-parameter (K2P) model by MEGA v7, and Structure v2.3.4 [46] was then used to display the genetic structures of different rodent species populations. The length of the burn-in period was set to 100,000 generations, and the number of Markov chain Monte Carlo repetitions was set to 200,000 generations. We chose the admixture model and the correlated allele frequencies model without prior group information during model setting. The clustering number of samples (K) was set from 1 to 8, repeating 10 times for each K. Structure Harvester (http://taylor0.biology.ucla.edu/structureHarvester/, accessed on 20 January 2019) was used to determine the optimal K value [47]. CLUMPP v1.1 [48] was used to calculate the Greedy algorithm for five repetitions, and graphs were drawn using Distruct v1.1 [49].

### 2.4. Echinococcus Species Infection Detection

For typical *Echinococcus* lesion samples, protoscoleces could be examined under a microscope [10]; however, protoscoleces could not be observed in typical lesion samples that were either too small (e.g., diameter < 1 mm) or calcified, or in liver tissue samples without obvious lesions. All lesion and liver tissue samples were therefore subjected to molecular analysis to confirm the presence of *Echinococcus* DNA.

DNA was extracted from lesion and liver tissue samples stored in 95% ethanol using a TIANamp Genomic DNA kit (Tiangen) following the manufacturer’s protocol. Two pairs of universal Taenidae primers and two pairs of *E. multilocularis*-, *E. shiquicus*-, and *E*. *granulosus*-specific primers (Table S1) were used for PCR to detect *Echinococcus* spp. DNA. A 25 µL reaction mixture contained 12.5 µL Premix Taq™ (Ex Taq™ Version 2.0) (Takara), 8.5 μL RNase-free water (Takara), 1 µL (10 µM) forward primer, 1 µL (10 µM) reverse primer, and 2 µL of sample DNA. The PCR cycler conditions for the amplification were 94 °C for 5 min (pre-denaturation), followed by 35 cycles of 94℃ for 30 s (denaturation), annealing for 45 s (setting the temperature for each primer pair following Table S1), 72 °C for 60 s (extension), and 72 °C for 10 min (final extension). The PCR products were sent to Sangon Biotech Co., Ltd. (Shanghai, China) for sequencing. BLAST was then used for sequence alignment to identify small mammals infected with *Echinococcus* species. The prevalence of each detected *Echinococcus* species in each small mammal host species was calculated [10].

### 2.5. Prevalence Evaluation

The population density (*D_i,j_*) of the *i*th host species and the prevalence (*P_i,j,k_*) of the *k*th *Echinococcus* species in the *i*th host species in the *j*th year were calculated according to *D_i,j_* = *n_i,j_*/*S_j_* and *P_i,j,k_* = *m_i,j,k_*/*n_i,j_*, respectively, where *n_i,j_* is the number of captured individuals of the *i*th host species in the *j*th year, *S_j_* is the number of quadrats in the *j*th year, and *m_i,j,k_* is the number of individuals of the *i*th host species detected with infection by the *k*th *Echinococcus* species in the *j*th year. The prevalence of the *Echinococcus* genus was calculated as the percentage of individuals infected with at least one *Echinococcus* species in a given year. The total prevalence of each *Echinococcus* species and the genus in each host small mammal species were calculated as the percentage of the total number of infected individuals among the total number of individuals checked during all four sampling years.

### 2.6. Statistical Analysis

We tested the difference in the dominance of each identified small mammal species according to its population density (number of captured individuals per quadrat) in different years by setting the small mammal species and time (in years) as two independent variables. Tukey’s honest significant difference method was used for the post hoc test. Small mammal species with significantly higher population densities were considered as dominant species. In this ANOVA design, the time variable was used as a constraint factor to control the order of the population density data of each species when testing for dominance. We further investigated significant changes in the relative importance of each species between years by performing *t*-tests for each species between years. 

We evaluated the prevalence (%) of *Echinococcus* in each host species using χ^2^ tests of independence to compare the the total prevalence of each *Echinococcus* species and the genus among small mammal species. We then evaluated the prevalence of *Echinococcus* species and the genus in terms of the correlation with the population size of each host species (assessed as the number of individuals of each small mammal species captured each year, ranked from small to large) using the Cochran–Armitage trend test [50]. 

All analyses were performed using R version 4.3.0 [51].

## 3. Results

### 3.1. Small Mammal Community Composition

A total of 1278 small mammals were captured during 2014–2019, including six species: *O. curzoniae* (*n* = 432), *Neodon fuscus* (Smokey vole, *n* = 643), *N. leucurus* (Blyth’s mountain vole, *n* = 120), *N. irene* (Irene’s mountain vole, *n* = 15), *Alexandromys limnophilus* (Lacustrine vole, *n* = 46), and *Cricetulus longicaudatus* (long-tailed dwarf hamster, *n* = 6), as well as 16 unidentified specimens with discordant dental characteristics (Figure S1). Detailed capture information is listed in Table 1. In addition, two incomplete rodent specimens without heads were excluded from the calculations and further analyses.

There were significant differences in density among the species based on the numbers of captured individuals in the same quadrats (*F* = 10.40, *p* < 0.001) (Table 1). The dominant small mammal species in terms of density was *N. fuscus*, followed by *O. curzoniae* (Table 1); however, the density of *O. curzoniae* was not significantly different from that of either the most abundant rodent species, *N. fuscus* (*p =* 0.772), or the rodent assemblage as a whole (density of all rodent individuals, *F* = 74.40, *p* = 0.092; Table 1). Although the dominance pattern remained stable across the years (*F* = 1.27, *p* = 0.315), the densities of *O. curzoniae*, the rodent assemblage as a whole, and the entire small mammal community decreased significantly with time (e.g., total specimens; Table 1).

### 3.2. Morphological Relationships among Rodent Species

**Figure 1:**
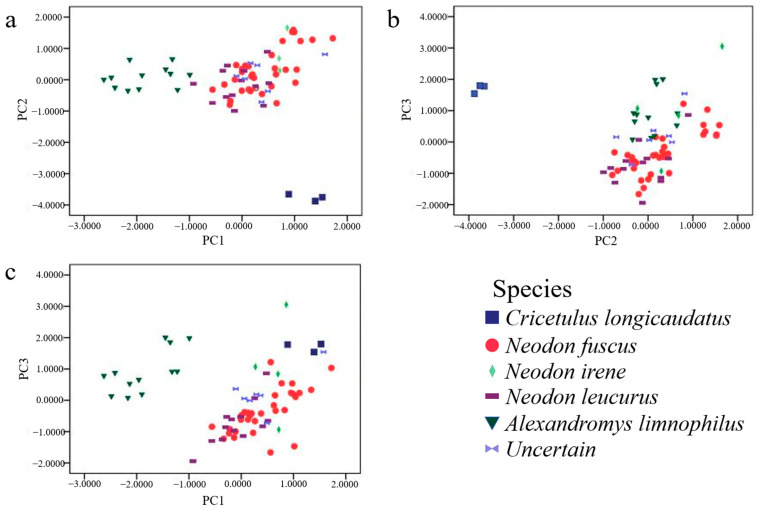
Scatter diagram of principal component (PCA) analysis of body and skull data of rodents. (**a**), Projections of individual specimen scores from principal component analysis on the 1st (PC1) and 2nd factors (PC2). (**b**), Projections of individual specimen scores from principal component analysis on PC2 and the 3rd factor (PC3). (**c**), Projections of individual specimen scores from principal component analysis on PC1 and PC3.

A total of 72 typical rodent specimens with complete external and cranial measurements, including 7 unidentified individuals (Table S4), were used for PCA. The eigenvalues of the first three principal components were ≥1, with 74.3% of the cumulative variance explained, and 14 out of the 20 morphological measurements were significant (absolute loading values ≥ 0.7) (Table S5). Spatial relationships among the 72 rodent specimens were presented using bivariate scatter plots according to their projected values in each of the three principal components. Only *C. longicaudatus* (Figure 1a,b) and *A. limnophilus* (Figure 1a,c) could be distinguished clearly from the complex of three *Neodon* species and unidentified specimens. 

### 3.3. Phylogenetic Relationships among Rodent Species

**Figure 2:**
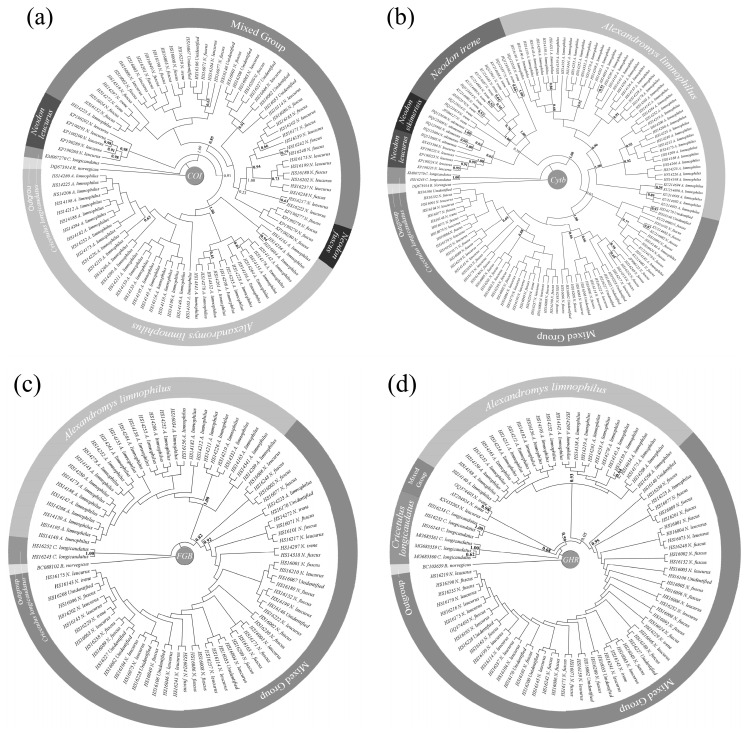
ML trees of rodent species related to Shiqu based on *COI* (**a**), *Cytb* (**b**), *FGB* (**c**), and *GHR* (**d**) gene sequences. Bootstrap values > 0.5 are in bold at related nodes, and values ≤ 0.5 but located at separated nodes for each group are displayed in unbolded font.

We examined the phylogenetic relationships among the detected rodent species based on 102 morphologically identified individuals, including 26 *N. fuscus*, 24 *N. leucurus*, 4 *N. irene*, 45 *A. limnophilus*, and 3 *C. longicaudatus*, and constructed topological trees. Ten unidentified individuals were also included in the trees to test their phylogenetic relationships with other known species. Together with the GenBank sequences, we obtained 135 *Cytb* gene sequences (98 from our specimens, 37 from GenBank), 94 *COI* gene sequences (84, 10), 83 *FGB* gene sequences, and 89 *GHR* gene sequences (82, 7). Sequences of the four tested *Rattus norvegicus* genes were obtained from GenBank as outgroups. Detailed information on the specimens, sequences, and accession numbers of the online data are given in Table S3. Regarding the tree construction, the best model for the *Cytb* and *COI* genes was HKY + I + G, while HKY + G was the best model for the *FGB* and *GHR* genes. Topological trees of the four gene segments calculated by the ML algorithm are presented in Figure 2, and similar Bayesian trees are presented in Figure S2. 

In the four final ML trees of the four genes, *C. longicaudatus* and *A. limnophilus* could be distinguished from other rodent species in Shiqu as solitary branches, while no topological structures could be distinguished for *N. fuscus*, *N. leucurus*, *N. irene*, or the 10 unidentified specimens in Shiqu in mixed groups (Figure 2). Analysis of the interspecific and intraspecific K2P distances confirmed that *N. fuscus*, *N. leucurus*, *N. irene*, and the unidentified specimens were closely related, with K2P distances < 0.005, which were no greater than the K2P distances between individuals of the same species (Table 2). In contrast, the K2P distances between *A. limnophilus* and *C. longicaudatus*, and between these two species and other rodent species were ≥50 times greater (Table 2).

**Figure 3:**
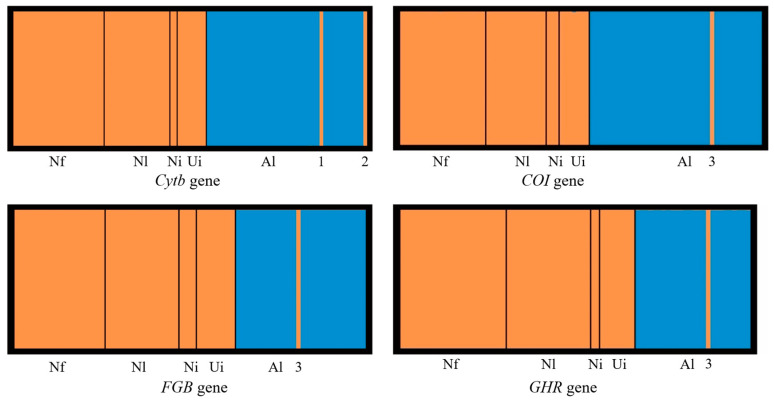
Population structure of rodent specimens in Shiqu based on *Cytb*, *COI*, *FGB*, and *GHR* genes. The structure analysis of all four genes suggested a significant K value of two for each. Group Orange was mainly composed of *Neodon fuscus* (Nf), *N. leucurus* (Nl), *N. irene* (Ni), and unidentified individuals (Ui). Group Blue was only composed of *A. limnophilus* (Al) specimens. Three morphologically identified *A. limnophilus* specimens, HS14140 (1), HS14182 (2), and HS14223 (3), were genetically included in Group Orange.

Structural analyses of the four genes were conducted based on 112 molecular rodent specimens collected in Shiqu County. We failed to retrieve *Cytb* data from our Shiqu *C. longicaudatus* specimens, and molecular data for Shiqu *C. longicaudatus* were therefore not available for all four genes, and this species was thus excluded from all four structure analyses. Each of the four final structure models gave a significant K value of two. Specimens identified morphologically as *N. fuscus*, *N. leucurus*, *N. irene*, and unidentified individuals were grouped together in all four gene structures (Group Orange, Figure 3). Although most specimens identified morphologically as *A. limnophilus* comprised one group (Group Blue, Figure 3), three individuals showed different genetic patterns. Two specimens (HS14140 and HS14182) showed similar *Cytb* genetic backgrounds to Group Orange, and one individual (HS14223) showed similar genetic backgrounds for the *COI*, *FGB*, and *GHR* genes to Group Orange. The structure results for these three individuals were inconsistent with the morphological species identification results but consistent with the phylogenetic tree results (Figure 2).

### 3.4. Echinococcus Prevalence in Small Mammals

*Echinococcus* protoscoleces were only detected in *N. fuscus* and *O. curzoniae*; in detail, four *N. fuscus* and one *O. curzoniae* in 2014, two *N. fuscus* and two *O. curzoniae* in 2015, and three *N. fuscus* and two *O. curzoniae* in 2016. However, molecular analysis detected *Echinococcus* DNA in samples from at least five out of the six small mammal species collected (Table 3).

There was no significant difference in prevalence among the three most abundant host species, *N. fuscus*, *O. curzoniae*, and *N. leucurus*, but the highest prevalence of *Echinococcus* species and the genus was detected in *A. limnophilus*. There was no significant difference in the prevalence of any of the *Echinococcus* species between *O. curzoniae* and the rodent assemblage (Table 3).

The Cochran–Armitage test for trends found inconsistent correlations between the three types of prevalence and the specimen size of the host species (Table 3). Regarding the prevalence of *E. multilocularis*, significant negative relationships were only detected in *O. curzoniae* (*Z* = 2.583, *p* = 0.005) and all small mammals (*Z* = 1.695, *p* = 0.045), with no significant relationships in other host species or the rodent assemblage; the prevalence of *E. shiquicus* was significantly positively related in *O. curzoniae* (*Z* = −1.884, *p* = 0.030), the rodent assemblage (*Z* = −1.899, *p* = 0.029), and all small mammals (*Z* = −3.040, *p* = 0.001). Regarding the prevalence of the *Echinococcus* genus, the only significant negative relationship was found in *O. curzoniae* (*Z* = 1.715, *p* = 0.043).

## 4. Discussion

Small mammals, as intermediate hosts of many *Echinococcus* species worldwide [10,13,52,53], have a diverse species composition and a wide geographic distribution on the Tibetan Plateau. According to Smith and Xie [34], over 100 recognized species of small mammals were distributed on the Tibetan Plateau, most belonging to the families Ochotonidae, Cricetidae, and Muridae, as putative intermediate hosts of *Echinococcus* species [53], and a phylogenetic study by Wang et al. suggested the local circulation system of *Echinococcus* on the Tibetan Plateau. How small mammals contribute to the establishment and dynamics of *Echinococcus* spp. circulation is a fundamental question relevant to the epidemiology of echinococcosis [10]; however, data on factors such as the exact distribution, population and community dynamics, and prevalence of these host species in specific localities, especially in endemic areas of the Tibetan Plateau, is currently lacking. This lack of data presents a major obstacle to understanding the ecology of the circulation of *Echinococcus* species and the mechanism of their co-evolution with host species in natural ecosystems in this region.

### 4.1. Species Composition of the Small Mammal Community

Information on the species composition of the small mammal community in Shiqu County has long been controversial, with a lack of relevant systematic research before the 21st century. *Neodon fuscus* was long considered to be distributed in Qinghai Province but not in Sichuan Province [33,34,54,55], and although Wang reported observations of *N. fuscus* at 11 sites in Shiqu County in 1959 [56], with no identified specimens, the distribution of this vole species in Shiqu County, Sichuan Province, remained unconfirmed. *Neodon fuscus* is morphologically similar to other sympatric vole species. To better understand the small mammal species composition, Raoul et al. studied small mammal species around Serxu Township (10 km west of our study area) in July 2001 and July 2002 and identified *C. kamensis*, *N. irene*, *N. leucurus*, *A. limnophilus*, *O. curzoniae*, and *C. cansus*, but not *N. fuscus* [21]. Similarly, He et al. carried out an epidemiological study of the prevalence of small mammals in Shiqu in 1997–1998 but failed to capture *N. fuscus* [57]. Qi et al., however, reported surveillance data for plague host species for 2001–2013, which indicated that *N. fuscus* might be the most abundant rodent species in Shiqu County [58], and Liu et al. confirmed the distribution of *N. fuscus* in Shiqu based on reliable morphological and phylogenetic methodologies [23]. In addition, *Eospalax baileyi* is also distributed in Shiqu County [59]. A summary of previous studies [21,23,58] and the current results (including [10]; Table 1) indicate that the small mammal population of the alpine meadows of Shiqu includes at least three pika species (*O*. *curzoniae*, *O*. *cansus*, and *O. erythrotis*) and eight rodent species (*N*. *fuscus*, *N leucurus*, *N. irene*, *A. limnophilus*, *C. longicaudatus*, *C. kamensis*, *Eozapus setchuanus*, and *E. baileyi*). Among these, our continuous surveillance data since 2014 indicated that *N. fuscus*, *O. curzoniae*, *N. leucurus*, *A. limnophilus*, *N. irene*, and *C. longicaudatus* were the six most frequently detected small mammal species (Table 1), all of which were theoretically valid intermediate host species for *Echinococcus*, although parasitological data for most of these species are still limited. 

### 4.2. Prevalence of Echinococcosis in Small Mammal Hosts

Most pathogens can infect multiple host species [60], which explains why most (ca. 60%) human pathogen species are zoonotic [61]. One reason why echinococcosis remains a serious epidemic in pastoral areas of the Tibetan Plateau is the presence of a diverse range of putative wildlife host species and the corresponding diverse routes of transmission. It is therefore important to understand the composition of these wildlife host species in remote areas, where basic research is still very weak, to provide an important foundation for integrated ecological management of echinococcosis. From a parasitological point of view (presence of protoscoleces), only a small number of wildlife species were confirmed as intermediate hosts of *E. multilocularis* and/or *E. shiquicus* in the Tibetan Plateau area. Regarding lagomorphs, *O. curzoniae* is the only confirmed common intermediate host species for *E. multilocularis* and *E. shiquicus* [20], and *Lepus oiostolus* is a confirmed intermediate host of *E. multilocularis* [62], while, among rodent intermediate host species, *N. fuscus* [10,18] and *N. irene* (previously *Microtus irene* [57]) are the only two confirmed host species for *E. multilocularis*. Although zokors (*E. baileyi*, recognized as a subspecies of *E*. *fontanierii* in [1]) have a vast distribution area in west China, the only infection data are from Ningxia for *E*. *fontanierii* [63]. The detection of *Echinococcus* DNA in at least five out of the six identified small mammal species in the current study (Table 3) suggests that the wild reservoir of *Echinococcus* spp. may include a more diverse range of small mammal intermediate host species. Longevities of pika and vole species are usually short in the wild, and they can become infected but be killed before protoscoleces form. This fact implies that a molecular methodology may be more efficient for finding potential infections than the traditional parasitological methodology. In addition, our preliminary analysis suggests that the correlation between the prevalence of different *Echinococcus* species and the population sizes of the different host species may vary, and many small mammal species are much rarer or more difficult to sample than the known and usually more common host species (Table 3). All of these limitations prevent a complete understanding of the composition and structure of the transmission system of echinococcosis. Further epidemiological studies of echinococcosis must therefore include more intensive routine population surveys of small mammal species in typical epidemic areas.

The importance of small mammal species in the cycling of *Echinococcus* spp. can be significantly affected by changes in population dynamics. These dynamics can vary greatly, from cyclical oscillations and stochastic changes to long-term high density or sudden extinction (as reviewed by Krebs [16]). To ensure the effective management of *Echinococcus* spp. transmission cycles, it is important to monitor the population dynamics of the host species closely. *Neodon irene* (previously *Microtus irene*), which was reported as a common and widespread species in Shiqu in the 1990s [13], was apparently much rarer, with small population sizes, as reported by Raoul et al. [21], Wang et al. [10], and in the current study (Table 1). Meanwhile, no *Echinococcus* spp. infections were detected in *N. irene* after 1997–1998 [57]. Thus, although trapping data may not reflect the exact abundance of each species, the smaller sample sizes for *N. irene* and other rarer small mammal host species make it difficult to detect typical lesions of *Echinococcus* infection or DNA and to make valid statistical comparisons. 

Unlike *N. irene*, *O. curzoniae* and *N. fuscus* were the two most abundant small mammal species (Table 1). Although the importance of rodent species as intermediate hosts of *E. multilocularis* on the Tibetan Plateau was noted as early as the 1990s [57], *O. curzoniae* has consistently been the most commonly examined small mammal host species [13]. Wang et al. noted that the importance of *N. fuscus* in the transmission of *E. multilocularis* and *E. shiquicus* should not be underestimated because of its similar prevalence and larger population size compared with *O. curzoniae* [10]. Meanwhile, *O. curzoniae* is considered the main target of poisoning population control programs because of its contribution to the degradation of the alpine meadow ecosystem on the eastern Tibetan Plateau [64,65,66]; for example, an *O. curzoniae* poisoning program was carried out in March, 2022 over a pastoral area of 92 km^2^, with the aim of killing 80,000 *O. curzoniae* individuals [67]. Such a significant change in the population density of *O. curzoniae* may result in increases in the populations and distributions of sympatric rodent species, such as *N. fuscus* [68]. The interspecific relationships among intermediate host species in the local small mammal community may thus be altered by human interventions, ultimately influencing the transmission patterns of *Echinococcus* spp. in the ecosystem. Zheng et al. confirmed that *O. curzoniae* was the main prey of the definitive wild host species, the Tibetan fox (*Vulpes ferrilata*), while *N. fuscus* only appeared in around 30% of fox feces [69]. The decline of the *O. curzoniae* population may thus force Tibetan foxes to increase predation on *N. fuscus* to compensate. Notably, an *O. curzoniae* individual may be 10 times heavier than an *N. fuscus* individual [34], and although there was no significant difference in the prevalence of *Echinococcus* spp. between *O. curzoniae* and *N. fuscus*, a Tibetan fox would have to prey on several *N. fuscus* to replace one *O. curzoniae*, thus increasing the risk of infection and the prevalence of echinococcosis in the Tibetan fox population. Therefore, changes in small mammal populations may affect the dietary behavior of their predator species, leading to variations in the infection burden in the wildlife reservoir and the consequent risk of echinococcosis in humans. In this study, the small mammal data were collected in an area without poisoning interference; although the population sizes of *O. curzoniae* and the rodent assemblage both decreased significantly (Table 1), data collected in just four years are not enough to confirm the population dynamic trends in these small mammals. In order to predict changes in the circulation of *Echinococcus* spp., it is therefore essential to understand the population dynamics of each relevant host species, especially small mammal intermediate host species, by long-term population surveillance. 

### 4.3. Effectiveness of Small Mammal Species Identification for Epidemiological Research

Intensive studies of the population dynamics and prevalence of *Echinococcus* spp. in small mammals require a clear knowledge of the taxonomy of the relevant species. In epidemiological studies, prevalence data usually need to be collected and reported promptly and an efficient and accurate taxonomic method for the identification of small mammal host species, especially rodent species, is therefore crucial. We tested the effectiveness of traditional quantitative and qualitative morphological and phylogenetic methods for identifying the five rodent species detected in this study. Specimens of *C. longicaudatus* and *A. limnophilus* could always be identified and distinguished from other species either by quantitative morphological measurements (e.g., PCA; Figure 1) or by each of the four phylogenetic analyses based on four genes (two mitochondrial and two nuclear) (Figure 2). By contrast, specimens of the three *Neodon* species could not be clearly distinguished from each other by either PCA or phylogenetic analyses (Figure 1 and Figure 2). Liu et al. revised the *Neodon* genus to include *N. fuscus* (formerly *Lasiopodomys fuscus*), *N. leucurus* (*Phaiomys leucurus*), and *N. irene* [22]. Their close genetic relationships were further confirmed by their interspecific K2P distances, which were no greater than the intraspecific distances (Table 2). DNA barcoding is considered a powerful and efficient method for species identification [70]; however, functional DNA barcodes are not available without effective gene loci, as found in relation to the identification of rodent species in Shiqu County. As a result, qualitative craniodental characteristics, such as the shape of the alveolar ridges and the position of the incisive foramen, remain the gold standard for rodent species identification, although the procedure is time consuming. Furthermore, although these qualitative characteristics identified most small mammal specimens, 16 rodent specimens remained unidentified due to their discordant dental characteristics (Figure S1), and three morphologically identified *A. limnophilus* specimens showed evidence of introgression from *Neodon* species (Figure 3). There is thus an urgent need for basic taxonomic studies of small mammal species assemblages in remote areas such as the Tibetan Plateau, especially to provide basic knowledge to support studies in other related fields, such as zoonotic disease epidemiology.

## 5. Conclusions

Lagomorphs and rodents are the main wild intermediate hosts of *E. multilocularis* and *E. shiquicus*. Understanding the species composition of these small mammals and the prevalence in typical endemic areas provides basic and essential knowledge of the epidemiology of echinococcosis. In this study, we reported the species composition of a small mammal community and the prevalence of *Echinococcus* spp. in Shiqu County, a typical endemic area of echinococcosis in the alpine pastoral area of the Tibetan plateau. Although the plateau pika (*O. curzoniae*) is still one of the main intermediate host species of the two *Echinococcus* species, the rodent assemblage composed of at least five recognized vole and hamster species may be more critical due to their larger numbers and even higher prevalence than the pika. The importance of rodent species in transmitting echinococcosis in the wildlife reservoir was long underestimated. To better understand the population dynamics and prevalence of *Echinococcus* spp. in these small mammals, explicit knowledge of the taxonomy of the relevant species is imperative. However, the fact that the species origin of specimens from the *Neodon* genus could not be clearly identified by current quantitative morphological and DNA barcoding methods indicated that effective and efficient molecular taxonomic tools are needed for epidemiological screening programs in genetically closely related wild host species.

## Figures and Tables

**Table 1:** Density (individuals per quadrat) of each small mammal species based on numbers of individuals captured in Shiqu County from 2014 to 2019 (number of individuals captured presented in parentheses).

	2014	2015	2016	2019	Total	Density Stability
*Neodon fuscus* ^ad^	36 (144)	68.3 (273)	19 (152)	12.3 (74)	29.2 (643)	*t* = 2.711, *p* = 0.073
*Ochotona curzoniae* ^abd^	33.8 (135)	17.5 (70)	20.8 (166)	10.2 (61)	19.6 (432)	***t*= 4.171, *p* = 0.025**
*Neodon leucurus* ^bc^	4 (16)	5 (20)	10.3 (82)	0.3 (2)	5.5 (120)	*t* = 2.374, *p* = 0.073
*Alexandromys limnophilus* ^c^	11 (44)	0	0.3 (2)	0	2.1 (46)	*t* = 1.036, *p* = 0.376
*Neodon irene* ^c^	1.5 (6)	0	0.3 (2)	1.2 (7)	0.7 (15)	*t* = 2.10, *p* = 0.1265
*Cricetulus longicaudatus* ^c^	0.3 (1)	0.5 (2)	0.4 (3)	0	0.3 (6)	*t* = 2.776, *p* = 0.069
Unidentified ^c^	0	0	1.3 (10)	1 (6)	0.7 (16)	-
Rodent assemblage ^d^	52.8 (211)	73.8 (295)	31.4 (251)	14.8 (89)	38.5 (846)	***t* = 4.235, *p* = 0.013**
Total specimens	86.5 (346)	91.3 (365)	52.1 (417)	25 (150)	58.1 (1278)	***t* = 4.09, *p* = 0.026**
Number of quadrats	4	4	8	6	22	

The two-way ANOVA analysis suggested significant differences in the size of small mammal populations. A Tukey honest significant difference post hoc test was conducted for *N. fuscus*, *O. curzoniae*, *N. leucurus*, *A. limnophilus*, *N. irene*, *C. longicaudatus*, unidentified individuals, and the rodent assemblage (all rodent specimens). Different superscripted letters indicate significant differences between the items compared, while the same superscripted letters suggest no significant difference. Bolded statistics in the density stability column show significant unstable (decreasing) results determined by *t*-tests.

**Table 2:** Intraspecific and interspecific K2P distances of the different species based on the *Cytb* gene, the *COI* gene, the *FGB* gene, and the *GHR* gene.

Species	K2P Distances of *Cytb* (above Diagnostic Line) and *COI* (under Diagnostic Line)						K2P Distances of *FGB* (above Diagnostic Line) and *GHR* (under Diagnostic Line)					
	*Neodon* *fuscus*	*Neodon leucurus*	*Neodon* *irene*	*Alexandromys* *limnophilus*	*Cricetulus* *longicaudatus*	Unidentified	*Neodon* *fuscus*	*Neodon* *leucurus*	*Neodon* *irene*	*Alexandromys* *limnophilus*	*Cricetulus* *longicaudatus*	Unidentified
*Neodon* *fuscus*	0.003	0.003	0.002	0.169	-	0.005	<0.001	<0.001	<0.001	0.015	0.088	<0.001
	0.003						0.001					
*Neodon leucurus*		0.003	0.002	0.170	-	0.004		<0.001	<0.001	0.015	0.088	<0.001
	0.003	0.003					0.001	0.001				
*Neodon* *irene*			0.001	0.169	-	0.005			<0.001	0.015	0.088	<0.001
	0.003	0.002	0.003				0.001	0.001	<0.001			
*Alexandromys limnophilus*				0.010	-	0.140				0.002	0.091	0.044
	0.141	0.141	0.142	0.018			0.044	0.044	0.044	0.003		
*Cricetulus longicaudatus*					-	0.262					<0.001	0.226
	0.261	0.261	0.260	0.238	-		0.227	0.226	0.225	0.221	<0.001	
Unidentified					-	0.006						<0.001
	0.004	0.004	0.004	0.171	-	0.003	0.001	0.001	0.001	0.015	0.088	0.001

**Table 3:** Prevalence (%) of *Echinococcus* species and genus in each small mammal species and its relation to the population of small mammals.

		Prevalence (No. Infected/No. Total, 95% Confidence Intervals)								
		*Neodon fuscus*	*Ochotona curzoniae*	*Neodon leucurus*	*Alexandromys limnophilus*	*Neodon irene*	*Cricetulus longicaudatus*	Unidentified	Rodent Assemblage	Small Mammals
*Echinococcus multilocularis*	2014	11.11 (16/144, 6.7–17.7)	1.48 (2/135, 0.3–5.8)	0 (0/16)	11.36 (5/44, 4.3–25.4)	0 (0/6)	0 (0/1)	-	9.95 (21/211, 6.41–15.01)	6.65 (23/346, 4.36–9.95)
	2015	0.37 (1/273, 0–2.4)	0 (0/70)	0 (0/20)	0	0	0 (0/2)	-	0.34 (1/295, 0.02–2.17)	0.27 (1/365, 0.01–1.75)
	2016	3.95 (6/152, 1.6–8.8)	1.81 (3/166, 0.5–5.6)	1.22 (1/82, 0.1–7.6)	0 (0/2)	0 (0/2)	0 (0/3)	10.00 (1/10, 0.5–45.9)	3.19 (8/251, 1.49–6.42)	2.64 (11/417, 1.39–4.81)
	2019	0 (0/74)	9.84 (6/61, 4.1–20.9)	0 (0/2)	0	0 (0/7)	-	0 (0/6)	0/89	4.00 (6/150, 1.64–8.89)
	Total	3.58 (23/643, 2.34–5.41) ^a^	2.55 (11/432, 1.35–4.65) ^a^	0.83 (1/120, 0.04–5.23) ^a^	10.87 (5/46, 4.07–24.36) ^b^	0 (0/15)	0 (0/6)	6.25 (1/16, 0.33–32.29)	3.55(30/846, 2.45–5.09) ^a^	3.21 (41/1279, 2.34–4.37)
	Cochran–Armitage test for trends	*Z* = 0.810, *p* = 0.209	**↓, *Z* = 2.583,** ***p* = 0.005**	*Z* = −0.618, *p* = 0.268	-	-	-	-	*Z* = 0.410, *p* = 0.341	**↓, *Z* = 1.695,** ***p* = 0.045**
*Echinococcus shiquicus*	2014	1.39 (2/144, 0.2–5.4)	3.70 (5/135, 1.4–8.9)	6.25 (1/16, 0.3–32.3)	11.36 (5/44, 4.3–25.4)	0 (0/6)	- (1/1)	-	4.27 (9/211, 2.10–8.21)	4.05 (14/346, 2.32–6.86)
	2015	3.66 (10/273, 1.9–6.8)	10.00 (7/70, 4.5–2.0)	0 (0/20)	0	0	0 (0/2)	-	3.39 (10/295, 1.73–6.34)	4.66 (17/365, 2.82–7.50)
	2016	1.97 (3/152, 0.5–6.1)	1.81 (3/166, 0.5–5.6)	1.22 (1/82, 0.1–7.6)	0 (0/2)	0 (0/2)	0 (0/3)	0 (0/10)	1.59 (4/251, 0.51–4.30)	1.68 (7/417, 0.74–3.58)
	2019	0 (0/74)	1.64 (1/61, 0.1–10.0)	0 (0/2)	-	0 (0/7)	-	0 (0/6)	0/89	0.67 (1/150, 0.04–4.22)
	Total	2.33 (15/643, 1.36–3.91) ^a^	3.70 (16/432, 2.20–6.07) ^a^	2.67 (2/120, 0.29–6.50) ^a^	10.87 (5/46, 4.07–24.36)^b^	0 (0/15)	16.67 (1/6, 0.88–63.52)	0 (0/16)	2.71 (23/846, 1.77–4.12)^a^	3.05 (39/1279, 2.21–4.19)
	Cochran–Armitage test for trends	**↑, *Z* = −1.884,** ***p* = 0.030**	*Z* = 0476, *p* = 0.317	*Z* = 0.939, *p* = 0.174	-	-	-	-	**↑, *Z* = −1.899,** ***p* = 0.029**	**↑, *Z* = −3.040,** ***p* = 0.001**
*Echinococcus* genus	2014	12.50 (18/144, 7.8–19.3)	5.19 (7/135, 2.3–1.1)	6.25 (1/16, 0.3–32.3)	22.73 (10/44, 12.0–38.2)	0 (0/6)	100.00 (1/1)	-	12.22 (30/211, 9.95–19.84)	10.69 (37/346, 7.73–14.55)
	2015	4.03 (11/273, 2.1–7.3)	10.00 (7/70, 4.5–20.1)	0 (0/20)	0	0	0 (0/2)	-	3.73 (11/295, 1.97–6.77)	4.93 (18/365, 3.03–7.82)
	2016	5.92 (9/152, 2.9–11.3	3.61 (6/166, 1.5–8.1)	2.44 (2/82, 0.4–9.4)	0 (0/2)	0 (0/2)	0 (0/3)	10.00 (1/10, 0.5–45.9)	4.78 (12/251, 2.61–8.41)	4.32 (18/417, 2.66–6.87)
	2019	0 (0/74)	9.84 (6/61, 4.1–20.9)	0 (0/2)	-	0 (0/7)	-	0 (0/6)	0/89	4.00 (6/150, 1.64–8.89)
	Total	5.91 (38/643, 4.27–8.10) ^a^	6.02 (26/432, 4.05–8.81) ^a^	2.50 (3/120, 0.65–7.68) ^a^	21.74 (10/46, 11.45–36.76)^b^	0 (0/15)	16.67 (1/6, 0.88–63.52)	6.25 (1/16, 0.33–32.29)	6.26 (53/846, 4.77–8.16) ^a^	6.18 (79/1279, 4.95–7.68)
	Cochran–Armitage test for trends	*Z* = −0.568, *p* = 0.285	**↓, *Z* = 1.715, *p* = 0.043**	*Z* = 0.410, *p* = 0.341	-	-	-	-	*Z* = −1.028, *p* = 0.152	*Z* = −1.131, *p* = 0.129

“Rodent assemblage” includes all the rodent specimens. “Small mammals” includes *O. curzoniae* and rodent species. Superscript letters indicate significant differences in the prevalence of different species as determined by χ^2^ tests of independence. There was a significant difference in prevalence between different species without the same letter superscripted. The differences in prevalence between species were only compared for *N. fuscus*, *O. curzoniae*, *N. leucurus*, *A. limnophilus*, and the rodent assemblage. *Neodon irene*, *C. longicaudatus*, and unidentified individuals were not included in the test because of their extremely small sample sizes. Relevant statistical values are presented in Table S6. The Cochran–Armitage test for trends was used to analyze the correlation between the prevalence and the population size of each host species or group. This analysis was only carried out in *N. fuscus*, *O. curzoniae*, the rodent assemblage, and small mammals. Significant correlations were marked in bold—represents that the data or statistics were not sufficient to conduct a test of correlation, ↑ represents a significant increase in prevalence with a bigger host species population size, and ↓ represents a significant decreasing correlation. Only one *C. longicaudatus* was captured and detected *E. shiquicus* infection in 2014, thus no prevalence and confidence intervals were calculated.

## Data Availability

Data will be made available on request.

## Supplementary Materials

The following supporting information can be downloaded at: https://www.mdpi.com/article/10.3390/pathogens13070558/s1, Table S1: The information of primers for PCR. Table S2: PCR reaction program. Table S3: Details of the sequences we downloaded from GenBank. Figure S1: The lower molars of the individuals showed different species characteristics on each side (1.6×). Table S4: The body and skull measurements of the rodent species. Table S5: Principal component analysis of the variables that responded to the morphological data of rodent species. Figure S2: BI tree of five rodent species in Shiqu based on *COI*, *Cytb*, *GHR*, and *FGB* gene sequences. Table S6: Statistical value of the χ^2^ test was used to compare the difference in the prevalence of *Echinococcus multiloculari*, *E. shiquicus*, and *Echinococcus* species between different small mammal species. References [22,23,71,72,73,74,75,76,77,78,79,80,81,82,83,84] are cited in the Supplementary Materials.
