# Supplementary material for: Species Composition of a Small Mammal Community and Prevalence of Echinococcus spp. in the Alpine Pastoral Area of the Eastern Tibetan Plateau
Source: Pathogens. 2024 Jul 2;13(7):558. doi: 10.3390/pathogens13070558 (PMC11280319; doi:10.3390/pathogens13070558)
Supplement: Supplementary file 1 [file pathogens-13-00558-s001.zip › pathogens-3074834-supplementary.pdf]

pathogens

MDPI

Supplementary Materials

Table S1. The information of primers for PCR.

Species	Genetic Marker	Primer	Primer Sequence	Annealing Temperature (°C)	Length (bp)	Reference
Rodent	*Cytb*	L14723<br>H15915	5′ - CCAATGACATGAAAAATCATCGTT - 3′<br>5′ - TCTCCATTTCTGGTTTACAAGAC - 3′	52	1140	[71]
Rodent	*COI*	COIF<br>COIR	5′ - TTGCAATTCGATGTGATT - 3′<br>5′ - ATGATGCTGGCTTGAAAC - 3′	50-60	1542	[22]
Rodent	*GHR*	GHR5<br>GHR6	5′ - GGCRTTCATGAYAACTACAAAC-CTGACYTC - 3′<br>5′ - GAGGAGAGGAACCTTCTTTTT-WTCAGGC -3′	53-61	921	[72]
Rodent	*FGB*	β17-mammL<br>βfib-mammU	5′ - ACCCCAGTAGTATCTGCCGTTT-GGATT - 3′<br>5′ - CACAACGGCATGTTCTTCAGCAC - 3′	60	204	[73]
Taeniidae	*COI*	COIF<br>COIR	5′ - TTGAATTTGCCACGTTTGAATGC - 3′<br>5′ - GAACCTAACGACATAACATAATGA - 3′	52	880	[74]
Taeniidae	*NDI*	JP11<br>JP12	5′ – AGATTCGTAAGGGGCCTAATA – 3′<br>5′ – ACCACTAACTAATTCACTTTC – 3′	55	500	[75]
Echinococcus multilocularis	*COI*	Em1<br>Em2	5′ – GTCATATTTGTTTAAGTATAAGTGG – 3′<br>5′ – CACTCTTATTTACACTAGAATTAA – 3′	52	243	[76]
Echinococcus multilocularis	*NDI*	EmF19/3<br>EmR6/1	5′ – TAGTTGTTGATGAAGCTTGTTG – 3′<br>5′ – ATCAACCATGAAAACACAT-ATACAAC – 3′	53	207	[77]
Echinococcus shiqucus	*COI*	Es1<br>Es2	5′ – GTTGGTTACGTTACCGGTT – 3′<br>5′ – TCTTATTAACATTTGAATTCAAC – 3′	52	420	[74]
Echinococcus shiqucus	*NDI*	EsF50<br>EsR73	5′ – TTATTCTCAGTCTCGTAAGGGTCCG – 3′<br>5′ – CAATAACCAACTACATCAATAATT – 3′	60	442	[77]
Echinococcus granulosus	*NDI*	Eg1F81<br>Eg1R83	5′ – GTTTTTGGCTGCCGCCAGAAC – 3′<br>5′ – AATTAATGGAAATAA-TAACAAACTTAATCAACAAT – 3′	62	226	[77]
Echinococcus granulosus	Genome	EgG1F<br>EgG1R	5′ – GAATGCAAGCAGCAGATG – 3′<br>5′ – GAGATGAGTGAGAAGGAGTG – 3′	55	113	[78]

Table S2. PCR reaction program.

Genetic Marker	Pre-Denaturation	Denaturation	Annealing	Extension	Cycles	Final-Extension
Cytb	9 °C 3 min	94 °C 30 s	55℃ 30s	72 °C 75s	35	725 min
COI	9 °C 3min	95 °C 45 s	605060s(Decrease by 0.5per cycle)	72 °C 90s	20	
		95 °C 45 s	5060s	72 °C 90s	20	7210 min
GHR	9 °C 5 min	95 °C 30 s	615330s(Decrease by 2every 5 cycles）	72 °C 60s	25	725 min
FGB	9°C 5 min	94 °C 60 s	6030s	72 °C 60s	30	727min

Table S3. Details of the sequences we downloaded from GenBank.

Accession number	Genetic marker	Species	Region	References/Author
KP190222	*Cytb*	*Neodon leucurus*	Lasa,Tibet,China	[22]
KP190223	*Cytb*	*Neodon leucurus*	Lasa,Tibet,China	[22]
KP190224	*Cytb*	*Neodon leucurus*	Lasa,Tibet,China	[22]
KP190225	*Cytb*	*Neodon leucurus*	Lasa,Tibet,China	[22]
KX455506	*Cytb*	*Neodon leucurus*	-	-
KU214679	*Cytb*	*Neodon fuscus*	Dari,Qinghai,China	-
KU214680	*Cytb*	*Neodon fuscus*	Dari,Qinghai,China	-
KU214681	*Cytb*	*Neodon fuscus*	Dari,Qinghai,China	-
KU214682	*Cytb*	*Neodon fuscus*	Dari,Qinghai,China	-
KU214683	*Cytb*	*Neodon fuscus*	Dari,Qinghai,China	-
JF906122	*Cytb*	*Neodon fuscus*	-	[79]
KU214684	*Cytb*	*Neodon irene*	Guide,Qinghai,China	-
KU214685	*Cytb*	*Neodon irene*	Guide,Qinghai,China	-
KU214686	*Cytb*	*Neodon irene*	Guide,Qinghai,China	-
KU214687	*Cytb*	*Neodon irene*	Guide,Qinghai,China	-
KU214688	*Cytb*	*Neodon irene*	Guinan,Qinghai,China	-
KU214689	*Cytb*	*Neodon irene*	Guinan,Qinghai,China	-
AM392370	*Cytb*	*Neodon irene*	-	[81]
JF906127	*Cytb*	*Neodon irene*	-	[79]
HQ123595	*Cytb*	*Neodon irene*	Luding,Sichuan,China	[23]
HQ123596	*Cytb*	*Neodon irene*	Luding,Sichuan,China	[23]
HQ123597	*Cytb*	*Neodon irene*	Luding,Sichuan,China	[23]
HQ123611	*Cytb*	*Neodon irene*	Dege,Sichuan,China	[23]
HQ123614	*Cytb*	*Neodon irene*	Dege,Sichuan,China	[23]
HQ123616	*Cytb*	*Neodon irene*	Dege,Sichuan,China	[23]
HQ123619	*Cytb*	*Neodon irene*	Yajiang,Sichuan,China	[23]
HQ123599	*Cytb*	*Neodon sikimensis*	Linzhi,Tibet,China	[23]
HQ123600	*Cytb*	*Neodon sikimensis*	Linzhi,Tibet,China	[23]
HQ123603	*Cytb*	*Neodon sikimensis*	Linzhi,Tibet,China	[23]
HQ123604	*Cytb*	*Neodon sikimensis*	Linzhi,Tibet,China	[23]
HQ123605	*Cytb*	*Neodon sikimensis*	Linzhi,Tibet,China	[23]
KU214690	*Cytb*	*Alexandromys limnophilus*	Menyuan,Qinghai,China	-
KU214691	*Cytb*	*Alexandromys limnophilus*	Menyuan,Qinghai,China	-
KU214692	*Cytb*	*Alexandromys limnophilus*	Menyuan,Qinghai,China	-
KU214693	*Cytb*	*Alexandromys limnophilus*	Menyuan,Qinghai,China	-
KU214694	*Cytb*	*Alexandromys limnophilus*	Menyuan,Qinghai,China	-
KM067270	*Cytb*	*Cricetulus longicaudatus*	Qinhai, China	[80]
DQ673914	*Cytb*	*Rattus norvegicus*	-	[81]
KP190277	*COI*	*Neodon fuscus*	Zhiduo,Qinghai,China	[22]
KP190278	*COI*	*Neodon fuscus*	Zhiduo,Qinghai,China	[22]
KP190279	*COI*	*Neodon fuscus*	Zhiduo,Qinghai,China	[22]
KP190280	*COI*	*Neodon fuscus*	Yushu,Qinghai,China	[22]
KP190288	*COI*	*Neodon leucurus*	Lasa,Tibet,China	[22]
KP190289	*COI*	*Neodon leucurus*	Lasa,Tibet,China	[22]
KP190290	*COI*	*Neodon leucurus*	Lasa,Tibet,China	[22]
KP190291	*COI*	*Neodon leucurus*	Lasa,Tibet,China	[22]
KP190292	*COI*	*Neodon leucurus*	Zhiduo,Qinghai,China	[22]

KM067270	*COI*	*Cricetulus longicaudatus*	Qinhai, China	[80]
DQ673914	*COI*	*Rattus norvegicus*	-	[81]
KX455565	*GHR*	*Neodon leucurus*	-	-
AY294924	*GHR*	*Neodon irene*	Yushu,Qinghai,China	[82]
GQ374493	*GHR*	*Neodon irene*	-	[79]
GQ374495	*GHR*	*Neodon fuscus*	-	[79]
MG685559	*GHR*	*Cricetulus longicaudatus*	Luun, central part, Mongolia	[83]
MG685560	*GHR*	*Cricetulus longicaudatus*	Qinghai, China	[83]
MG685561	*GHR*	*Cricetulus longicaudatus*	Tuva, Russia	[83]
BC103659	*GHR*	*Rattus norvegicus*	-	[84]
BC088102	*FGB*	*Rattus norvegicus*	-	[84]

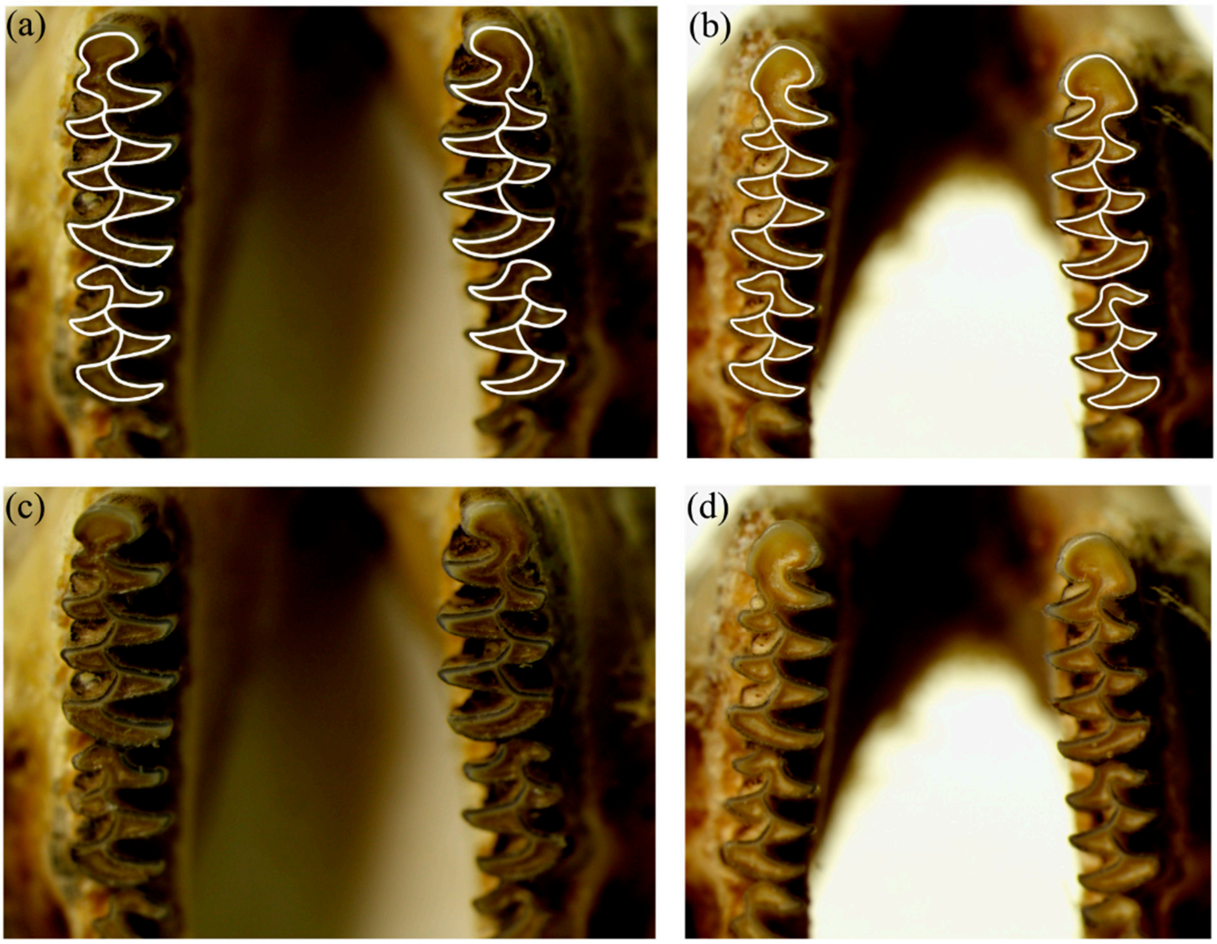

Figure S1. The lower molars of the individuals showed different species characteristics in each side （1.6×） . Figure S1 a and S1b showed the morphological characteristics of *N. fuscus* on the left lower molars and *N. leucurus* on the right. Original photos were presented in S1c and S1d.

Table S4. The body and skull measurements of the rodent species.

Measurements	*Neodon fuscus* *n* = 33	*Neodon leucurus* *n* = 13	*Neodon irene* *n* = 4	Unidentifed *n* = 7	*Alexandromys limnophilus* *n* = 12	*Cricetulus longicaudatus* *n* = 3
WT	42.62(26.30–76.60)	35.03(24.60–52.40)	52.98(35.90–70.90)	39.90(28.80–63.90)	25.54(18.00–45.50)	30.23(25.70–36.70)
HBL	110.27(94.53–135.13)	104.09(92.94–122.83)	115.13(99.12–130.34)	105.60(98.09–117.2)	95.90(79.77–115.07)	96.56(88.23–106.33)
TL	31.36(24.68–36.67)	29.52(24.16–33.81)	33.71(32.34–35.57)	30.69(25.67–36.62)	31.59(27.41–36.55)	32.56(29.86–35.40)
EL	12.34(11.01–14.52)	11.73(8.93–14.19)	12.80(11.38–14.10)	13.43(13.10–13.76)	13.84(12.52–16.90)	17.80(17.69–17.90)
FFL	12.14(10.34–13.24)	12.16(11.25–13.14)	12.54(11.43–13.07)	12.26(12.12–12.40)	8.95(8.24–9.67)	9.90(9.31–10.48)
SH	35.88(30.91–39.78)	35.39(31.49–39.35)	40.39(32.99–47.62)	40.63(36.84–44.42)	36.96(33.93–40.55)	39.40(38.79–40.00)
HLL	37.53(30.98–43.96)	37.09(30.31–41.39)	45.54(35.41–55.41)	38.70(38.13–39.26)	42.82(38.40–46.74)	39.30(38.24–40.35)
HFL	21.19(19.15–25.50)	20.24(19.22–22.22)	20.87(19.98–22.30)	20.52(20.05–20.98)	17.87(16.35–19.16)	17.95(17.86–18.04)
GSL	28.29(25.92–31.87)	26.94 (24.84–29.14)	28.46(27.11–29.93)	28.16(26.74–31.49)	24.75(23.43–26.01)	27.73(26.86–28.41)
ZB	16.68(14.93–19.16)	15.69(14.14–17.83)	17.13(15.97–17.78)	16.13(13.95–18.11)	14.09(13.02–16.26）	14.25(14.25–14.25)
BRO	5.31(4.83–6.11)	5.07(4.72–5.49)	5.41(5.19–5.74)	5.28(5.03–5.87)	4.55(4.20–5.03)	5.09(4.83–5.55)
LMxt	6.03(5.62–6.90)	5.68(5.34–6.32)	6.13(5.81–6.62)	5.93(5.59–6.39)	5.86(5.53–6.27)	4.13(4.07–4.22)
ORL	10.09(9.14–11.34)	9.68(9.21–10.84)	10.11(9.40–10.64)	10.04(9.47–10.72)	9.01(8.53–9.69)	9.15(8.27–10.02)
BM1	1.34(1.22–1.50)	1.31(1.23–1.38)	1.37(1.18–1.55)	1.36(1.25–1.47)	1.28(1.19–1.47)	1.19(1.13–1.23)
M1M	5.59(5.03–6.16)	5.39(5.16–5.85)	5.78(5.52–6.08)	5.57(5.36–5.91)	4.89(4.71–5.27)	5.66(5.57–5.82)
LIF	4.83(4.05–6.09)	4.72(4.18–5.44)	5.09(4.78–5.68)	4.91(4.30–5.39)	4.13(3.54–4.71)	5.20(4.98–5.57)
BIF	1.10(0.66–1.34)	0.97(0.81–1.16)	1.18(1.10–1.27)	1.03(0.87–1.33)	0.88(0.72–1.09)	1.47(1.22–1.68)
SKH	11.65(10.79–12.50)	11.24(10.61–12.09)	12.05(11.70–12.30)	11.88(11.20–12.74)	10.66(9.94–11.53)	10.34(9.94–10.82)
BB	13.81(12.38–15.12)	13.42(12.36–14.19)	13.81(13.26–14.13)	14.34(13.21–16.83)	11.94(11.06–13.57)	11.52(11.23–11.70)
LMbT	5.88(5.24–6.72)	5.59(5.01–6.11)	5.92(5.65–6.39)	5.70(5.45–6.25)	5.68(5.22–5.84)	4.18(4.14–4.20)

The unit of WT is g and the units of other measurements are mm. Unidentifed represents the individuals whose morphological characteristics of bilateral lower molars on both sides are inconsistent. Measurements: WT (weight), HBL (head and body length), TL (tail length), EL (ear length), FFL (forefoot length), SH (shoulder height), HLL (hind leg length), HFL (hind foot length), GSL(greatest skull length), ZB(zygomatic breadth), BRO(rostrum breadth), LMxT(length of maxillary toothrow), ORL(orbital length), BM1(breadth of first maxillary molars), M1M(external alveolar breadth), LIF(length of incisive foramen), BIF(breadth of incisive foramen), SKH(skull height), BB(breadth of braincase), LMbT(length of the mandibular toothrow).

Table S5. Principal components analysis of the variables that responded to the morphological data of rodent species.

Variable	Component		
	PC1	PC2	PC3
WT	**0.708**	0.553	0.294
HBL	0.643	0.538	0.246
TL	0.280	0.244	0.593
EL	0.070	−0.431	**0.742**
FFL	**0.700**	0.266	−0.466
SH	0.165	0.056	**0.730**
HLL	−0.199	0.182	**0.780**
HFL	0.615	0.307	−0.440
GSL	**0.878**	0.365	0.143
ZB	**0.722**	0.560	−0.015
BRO	**0.860**	0.353	0.011
LMxT	0.081	**0.923**	0.035
ORL	**0.710**	0.583	0.032
BM1	0.314	0.655	0.071
M1M	**0.851**	0.237	0.015
LIF	**0.735**	−0.141	0.136
BIF	**0.709**	−0.140	0.324

SKH	0.628	0.593	−0.051
BB	0.665	0.590	−0.140
LMbT	0.103	**0.929**	−0.010
Eigenvalues	9.931	2.822	2.103
Percent of variance (%)	49.657	14.112	10.513
Accumulative contribution rate (%)	49.657	63.769	74.282

Loadings of variables with absolute values > 0.6 were marked in bold. Measurements: WT (weight), HBL (head and body length), TL (tail length), EL (ear length), FFL (forefoot length), SH (shoulder height), HLL (hind leg length), HFL (hind foot length), GSL(greatest skull length), ZB(zygomatic breadth), BRO(rostrum breadth), LMxT(length of maxillary toothrow), ORL(orbital length), BM1(breadth of first maxillary molars), M1M(external alveolar breadth), LIF(length of incisive foramen), BIF(breadth of incisive foramen), SKH(skull height), BB(breadth of braincase), LMbT(length of the mandibular toothrow).

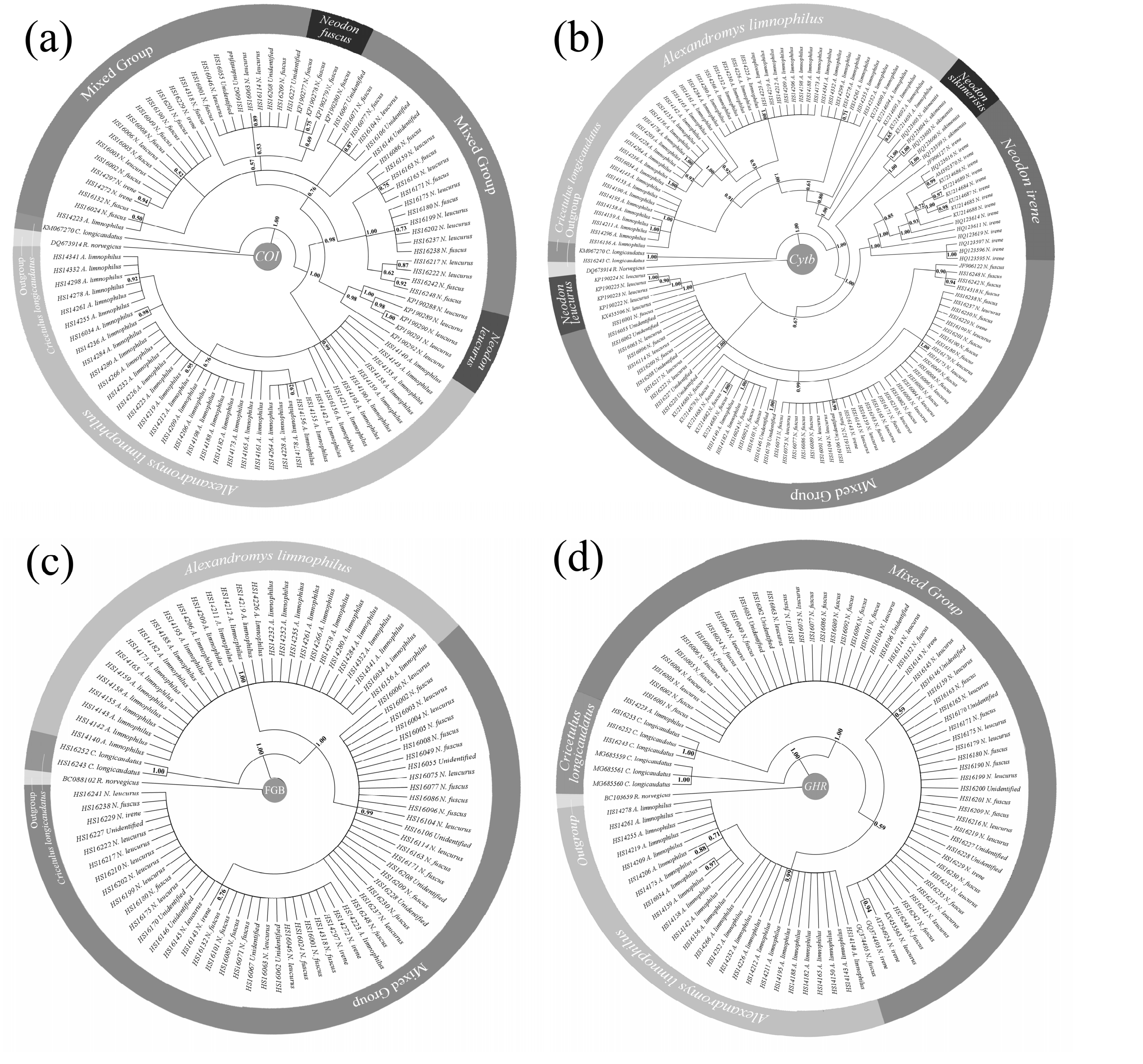

Figure S2. BI tree of five rodent species in Shiqu based on *COI* (**a**), *Cytb* (**b**), *GHR* (**d**) and *FGB* (**c**) gene sequences.

Table S6. Statistical value of the χ^2 test was used to compare the difference in the prevalence of *Echinococcus multilocularis* (Gray font, Lower-left), *E. shiquicus* (Dark font, Lower-left) and *Echinococcus* species (Dark font, Upper-right) between different small mammal species.

	Neodon fuscus	***Ochotona curzoniae***	***Neodon leucurus***	***Alexandromys limnophilus***	**Rodentia**
Neodon fuscus		χ^2 = 0.000, *P* = 1.000	χ^2 = 1.690, *P* = 0.194	χ^2 = 14.244, *P* < 0.001	χ^2 = 0.030, *P* = 0.862
Ochotona curzoniae	χ^2 = 0.951, *P* = 0.442<br>χ^2 = 1.279, *P* = 0.258		χ^2 = 1.682, *P* = 0.195	χ^2 = 12.582, *P* < 0.001	χ^2 = 0.003, *P* = 0.960
Neodon leucurus	χ^2= 1.679, *P* = 0.195<br>χ^2 = 0.014, *P* = 0.907	χ^2 = 0.615, *P* = 0.433<br>χ^2 = 0.674, *P* = 0.412		χ^2 = 14.491, *P* < 0.001	χ^2 = 2.082, *P* = 0.149
Alexandromys limnophilus	χ^2 = 4.135, *P* = 0.042<br>χ^2 = 8.278, *P* = 0.004	χ^2 = 6.515, *P* = 0.011<br>χ^2 = 3.520, *P* = 0.061	χ^2 = 6.949, *P* = 0.008<br>χ^2 = 4.880, *P* = 0.027		χ^2 = 13.645, *P* < 0.001
Rodent assemblage	χ^2 = 0.000, *P* = 1.000<br>χ^2 = 0.091, *P* = 0.763	χ^2 = 0.627, *P* = 0.429<br>χ^2 = 0.634, *P* = 0.426	χ^2 = 1.693, *P* = 0.193<br>χ^2 = 0.138, *P* = 0.710	χ^2 = 4.416, *P* = 0.037<br>χ^2 = 7.041, *P* = 0.008	

References:

22. Liu, S.Y.; Jin, W.; Liu, Y.; Murphy, R.W.; Lv, B.; Hao, H.B.; Liao, R.; Sun, Z.Y.; Tang, M.K.; Chen, W.C.; et al. Taxonomic position of Chinese voles of the tribe Arvicolini and the description of 2 new species from Xizang, China. *Journal of Mammalogy* **2017**, 98, 166-182. https://doi.org/10.1093/jmammal/gyw170.
23. Liu, S.Y.; Sun, Z.Y.; Liu, Y.; Wang, H.; Guo, P.; Murphy, R.W. A new vole from Xizang, China and the molecular phylogeny of the genus *Neodon* (Cricetidae: Arvicolinae). *Zootaxa* **2012**, 3235, 1-22. https://doi.org/10.11646/zootaxa.3235.1.1.
71. Ducroz, J.F.; Volobouev, V.; Granjon, L. An Assessment of the Systematics of Arvicanthine Rodents Using Mitochondrial DNA Sequences: Evolutionary and Biogeographical Implications. *Journal of Mammalian Evolution* **2001**, 8, 173-206. https://doi.org/10.1023/A:1012277012303.
72. Galewsk, T.; Tilak, M.; Sanchez, S.; Chevret, P.; Paradis, E.; Douzery, E.J. The evolutionary radiation of Arvicolinae rodents (voles and lemmings): relative contribution of nuclear and mitochondrial DNA phylogenies. *BMC Ecology and Evolution* **2006**, 6, 80. https://doi.org/10.1186/1471-2148-6-80.
73. Matocq, M.D.; Shurtliff, Q.R.; Feldman, C.R. Phylogenetics of the woodrat genus *Neotoma* (Rodentia: Muridae): Implications for the evolution of phenotypic variation in male external genitalia. *Molecular Phylogenetics and Evolution* **2007**, 42, 637-652. https://doi.org/10.1016/j.ympev.2006.08.011.
74. Jiang, W.B.; Liu, N.; Zhang, G.T.; Renqing, P.C.; Xie, F.; Li, T.Y.; Wang, Z. H.; Wang, X.M. Specific detection of *Echinococcus* spp. from the Tibetan fox (*Vulpes ferrilata*) and the red fox (*V. vulpes*) using copro-DNA PCR analysis. *Parasitology Research* **2012**, 111, 1531-1539. https://doi.org/10.1007/s00436-012-2993-8.
75. Gasser, R.B.; Zhu, X.; McManus, D.P. NADH dehydrogenase subunit 1 and cytochrome c oxidase subunit I sequences compared for members of the genus *Taenia* (Cestoda). *International Journal for Parasitology*, **1999**, 29, 1965-1970. https://doi.org/10.1016/s0020-7519(99)00153-8.

76. Nakao M.; Li T.Y.; Han X.M.; Ma X.M.; Xiao N.; Qiu J.M.; Wang H.; Yanagida T.; Mamuti W.; Wen H. et al. Genetic polymorphisms of Echinococcus tapeworms in China as determined by mitochondrial and nuclear DNA sequences. *International Journal for Parasitology* **2010**, 40, https://doi.org/379-385. 10.1016/j.ijpara.2009.09.006.
77. Boufana B.; Qiu J.M.; Chen X.W.; Budke C.M.; Campos-Ponce M.; Craig P.S. First report of Echinococcus shiquicus in dogs from eastern Qinghai–Tibet plateau region, China. *Acta Tropica* **2013**, 127, 21-24. https://doi.org/10.1016/j.actatropica.2013.02.019.
78. Abbasi I.; Branzburg A.; Campos-Ponce M.; Abdel H.S.; Raoul F.; Craig P.S.; Hamburger J. Copro-diagnosis of *Echinococcus granulosus* infection in dogs by amplification of a newly identified repeated DNA sequence. *The American Journal of Tropical Medicine and Hygiene* **2003**, 69, 324-330. https://doi.org/10.4269/ajtmh.2003.69.324.
79. Chen, W.C.; Hao, H.B.; Sun, Z.Y.; Liu, Y.; Liu, S.Y.; Yue, B.S. Phylogenetic position of the genus *Proedromys* (Arvicolinae, Rodentia): Evidence from nuclear and mitochondrial DNA. *Biochemical Systematics and Ecology* **2012**, 42, 59-68. https://doi.org/10.1016/j.bse.2012.01.002.
80. Zhang, Z.Q.; Sun, T.; Kang, C.L.; Liu, Y.; Liu, S.Y.; Yue, B.S.; Zeng, T. The complete mitochondrial genome of lesser long-tailed Hamster *Cricetulus longicaudatus* (Milne-Edwards, 1867) and phylogenetic implications, *Mitochondrial DNA A* **2014**, 27, 1303-1304. https://doi.org/10.3109/19401736.2014.945567.
81. Schlick, N.E.; Jensen-Seaman, M.I.; Orlebeke, K.; Kwitek, A.E.; Jacob, H.J.; Lazar, J. Sequence analysis of the complete mitochondrial DNA in 10 commonly used inbred rat strains. *American Journal of Physiology-Cell Physiology* **2006**, 291, C1183-C1192. https://doi.org/10.1152/ajpcell.00234.2006.
82. Steppan, S.; Adkins, R.; Anderson, J. Phylogeny and divergence-date estimates of rapid radiations in muroid rodents based on multiple nuclear genes. *Systematic Biology* **2004**, 53, 533-53. https://doi.org/10.1080/10635150490468701.
83. Lebedev, V.S.; Bannikova, A.A.; Neumann, K.; Ushakova, M.V.; Ivanova, N.V.; Surov, A.V. Molecular phylogenetics and taxonomy of dwarf hamsters *Cricetulus* Milne-Edwards, 1867 (Cricetidae, Rodentia): description of a new genus and reinstatement of another. *Zootaxa* **2018**, 4387, 331-349. https://doi.org/10.11646/zootaxa.4387.2.5.
84. Strausberg, R.L.; Feingold, E.A.; Grouse, L.H.; Derge, J.G.; Klausner, R.D.; Collins, F.S.; Wagner, L.; Shenmen, C.M.; Schuler, G.D.; Altschul, S.F.; et al. Mammalian Gene Collection Program Team. Generation and initial analysis of more than 15,000 full-length human and mouse cDNA sequences. *PNAS* **2002**, 99, 16899-903. https://doi.org/ 10.1073/pnas.242603899.
